# Construction of a Yeast Protein-Chitooligosaccharide W/O/W Emulsion System for Carrying and Stabilization of Betacyanins

**DOI:** 10.3390/foods14081337

**Published:** 2025-04-13

**Authors:** Yichen Li, Jiaqi Ding, Yaxin Wu, Shihao Sun, Demei Meng, Chunkai Gu, Rui Yang

**Affiliations:** 1 State Key Laboratory of Food Nutrition and Safety, College of Food Science and Engineering, Tianjin University of Science & Technology, Tianjin 300457, China; 2 Beijing Life Science Academy, Beijing 102209, China

**Keywords:** Yeast protein, Chitooligosaccharides, W/O/W emulsions, Micromorphological characteristics, Stability

## Abstract

Natural pigments like betacyanins are highly unstable under heat, light, acid, and alkaline conditions. Yeast protein (YP) is a promising substitute protein, while chitooligosaccharides (COS) are water-soluble alkaline polysaccharides. Water-in-oil-in-water (W_1_/O/W_2_) emulsions, with two-membrane, three-phase structure, can serve as effective carriers for stabilizing pigments. In this study, YP-COS complexes formed through electrostatic interactions were used as hydrophilic emulsifiers to create betacyanin-coated W_1_/O/W_2_ emulsions. The W_1_/O colostrum was designed to make up 30%, 70%, and 90% of the emulsion (*v*/*v*)and the W_2_ was designed by the complexes with three concentrations of YP (2%, 1.25% and 0.5%, *w*/*v*)-COS (6%, 3.75% and 1.5%, *w*/*v*). The optimal formulation was determined through comprehensive evaluation of micromorphological characteristics, particle size, zeta potential and creaming index, ultimately yielding a system comprising YP (2%)-COS (6%) and 90% W_1_/O colostrum. Moreover, the W_1_/O/W_2_ emulsion system significantly improved the betacyanins retention under thermal treatment, photolytic exposure, pH gradients, and extended storage compared to the betacyanin aqueous solution (*p* < 0.05). In vitro digestion tests showed the emulsion retained 58.39% of betacyanins, while the betacyanin aqueous solution retained only 41.42%, demonstrating the emulsion’s ability to delay the betacyanins release, offering new insights for using YP-COS complexes in food production and other fields.

## 1. Introduction

Betacyanins are natural pigments primarily comprising yellow-orange betaxanthin and reddish-purple betaxanthin. As functional food natural colorants, betacyanins have high antioxidant activity and many health benefits, including anti-cancer, anti-diabetic and anti-inflammatory activity [1]. Despite these advantages, the practical application of these phytochemicals is substantially constrained by their inherent chemical instability under various environmental conditions [2]. Current research on enhancing betacyanin stability through emulsion systems remains limited, with few systematic studies addressing this specific application. Emulsions are recognized as effective and widely utilized packaging systems that improve the bioaccessibility of bioactive nutrients [3]. The conventional emulsions (O/W emulsions and W/O emulsions) frequently exhibit limitations due to single-phase load constraints and inadequate oxidation resistance, resulting in suboptimal delivery [4]. Water-in-oil-in-water (W/O/W) emulsions, characterized by their unique bilayered architecture and triphasic compartmentalization, demonstrate enhanced dual-phase loading capacity for hydrophilic and lipophilic bioactive compounds [5]. This structural advantage positions W/O/W emulsions as advanced delivery platforms for multifunctional food applications, particularly in flavor modulation and micronutrient enrichment through core-shell encapsulation mechanisms. Nevertheless, the increased complexity of these multiphase systems renders them more susceptible to destabilization phenomena compared to W/O emulsions [6]. It is mainly manifested in physical instability due to the passage of molecules from the inner to outer phases, which presents ongoing challenges [7]. Extant research has established foundational parameters for betacyanin encapsulation in W/O/W emulsions (e.g., particle size distribution and surface charge characteristics). However, the study remains a critical knowledge gap regarding the system’s capacity to mitigate betacyanin degradation under real-world stressors, including photolytic, thermal, pH fluctuation, and storage time, etc. [8]. Recent advances in W/O/W emulsion stabilization strategies have prioritized interfacial engineering of the external aqueous phase. Notably, bio-macromolecular stabilizers derived from natural sources—particularly protein-polysaccharide complexes have emerged as promising emulsifiers and stabilizers for the field of W/O/W emulsions.

Biopolymer particles formed through non-covalent interactions within protein-polysaccharide complexes have garnered considerable attention due to their straightforward preparation methods and inherent properties derived from natural materials [9]. This paradigm is exemplified by the work of Zhuang et al., who engineered a W/O/W emulsion gel system incorporating grape seed proanthocyanidins, employing whey protein isolate reinforced by konjac glucomannan conjugation [10]. Yeast protein (YP), a high-quality alternative protein, offers various functional benefits in the food industry [11,12]. Chitooligosaccharides (COS), derived from chitosan degradation, exhibit superior water solubility and enhanced antibacterial and anti-inflammatory activities [13,14]. Previous studies have focused on the interaction between COS and proteins such as whey protein and soy protein isolate [15,16]. Meanwhile, COS also serve as excellent hydrophilic emulsifiers, forming non-covalent complexes with proteins, including casein and whey protein, to form non-covalent bonding complexes to stabilize W/O/W emulsions [17]. However, the structure-function relationship of YP-COS complexes in the W_1_/O/W_2_ emulsions remains unexplored.

This study engineers an optimized W_1_/O/W_2_ emulsion system stabilized by YP-COS complexes, aiming to concurrently enhance colloidal stability and betacyanin bioaccessibility. The double emulsion architecture is fabricated via a sequential two-stage emulsification process. To assess the optimal emulsion systems, the study evaluates emulsion structure elucidation, droplet sizes, zeta potential, and creaming index quantification. After determining the optimal emulsion systems, the W_1_/O/W_2_ emulsions are compared with betacyanin aqueous solution under various conditions, including thermal treatment, photolytic exposure, pH gradients, and extended storage. Bioaccessibility quantification is conducted through standardized in vitro gastrointestinal models. This work advances food-grade encapsulation technologies by establishing structure-function relationships between composite stabilizers and emulsion performance, providing a scalable platform for natural pigment applications in functional foods and nutraceuticals. Meanwhile, it also provides new insights into pigment embedding systems by protein-polysaccharide complexes.

## 2. Materials and Methods

### 2.1. Materials

Yeast protein (YP), sourced from Hubei Angel Yeast Co., Ltd. (Yichang, China), had a content of 75% (*w*/*w*) as measured by Lowry’s method. Betacyanins (molecular weight: 550.475) and polyglycerol polyricinoleate (PGPR) were acquired from Shanghai Macklin Biochemical Technology Co., Ltd. (Shanghai, China). Chitooligosaccharides (COS, 97% deacetylation) came from Zhejiang Golden Shell Biochemical Co., Ltd. (Taizhou, China). Edible-grade rapeseed oil was supplied by Tianjin Kerry Grain and Oil Co., Ltd. (Tianjin, China). PBS buffer (dry powder) was obtained from Wuhan Sevier Biotechnology Co., Ltd. (Wuhan, China). Anhydrous ethanol (99.5% purity) and n-hexane (99% purity) were purchased from Shanghai Macklin Biochemical Technology Co., Ltd. (Shanghai, China). Additional reagents, including sodium hydroxide and concentrated hydrochloric acid, were of analytical grade.

### 2.2. Preparation of YP-COS Complex

A defined quantity of YP was solubilized in phosphate-buffered saline (PBS) (0.01 M, pH 7.0) to prepare a YP solution with a concentration of 4% (*w*/*v*). This solution underwent continuous agitation using a magnetic stirrer (JOANLAB, MMS6Pro, Huzhou, China) at 400 rpm for 30 min at ambient temperature (25 °C) to facilitate the effective removal of impurities. In parallel, an appropriate amount of COS was dissolved in an identical PBS buffer to achieve a 12% (*w*/*v*) stock solution. Equimolar volumes of YP and COS solutions were combined at a 1:3 mass ratio, generating a composite system containing 2% (*w*/*v*) YP and 6% (*w*/*v*) COS. The mixture underwent immediate ultrasonication using an ultrasonic cell disruptor (Scientz-IID, NingBo Scientz Bio. Co, Ningbo, China) under light-protected conditions at 4 °C (200 W, 15 min) [18]. The complexes were formed by stirring the mixture on the magnetic stirrer (JOANLAB, MMS6Pro, Huzhou, China) at 400 rpm for an additional 15 min to ensure complete dissolution and homogeneity. Two distinct sample preparations of YP-COS complexes were established: (1) a group maintaining the original concentration (YP (2%)-COS (6%), *w*/*v*), and (2) two dilution groups of samples subjected to proportional dilution according to varying concentrations. The dilution process yielded two distinct formulations: one producing a YP (1.25%)-COS (3.75%) (*w*/*v*) complex and the other yielding a YP (0.5%)-COS (1.5%) (*w*/*v*) complex upon completion of the dilution procedures. Ultimately, three sets of YP-COS complex formulations with different concentrations were obtained as the outer aqueous phase (W_2_).

### 2.3. Preparation of the W_1_/O Colostrum

The oil phase was formulated by blending rapeseed oil with polyglycerol polyricinoleate (PGPR), which was frequently employed as a crucial ingredient in stable gel emulsifiers. Betacyanins had excellent water solubility, allowing them to be dissolved directly to form solutions. Initially, the PGPR was sequentially added to the oil at three concentration levels (0.02%, 0.025%, and 0.03%, *v*/*v*) with concentrations calculated based on total lipid phase volume. Then, the PGPR was stirred with rapeseed oil for 30 min as the oil phase, respectively, using a magnetic stirrer (JOANLAB, MMS6Pro, Huzhou, China) set at 400 rpm. Subsequently, 10 mg of betacyanins were accurately weighed and dissolved in 5 mL PBS (0.01 M, pH 7.0) to obtain 2 mg/mL betacyanin aqueous solutions, serving as the inner water phase (W_1_) for the W_1_/O colostrum formulation. Next, varying volumes of the inner water phases were added into the prepared oil phase at ratios of W_1_ to oil phase equal to 1:9, 3:7, and 5:5. Finally, the W_1_ and oil phase were mixed through a variable-speed homogenizer (a rotor-stator device) (Yixin, FSH-2A, Changzhou, China) operated at 10,000 rpm for 2 min for preparing the W_1_/O colostrum with distinct water (W_1_)/oil ratios.

### 2.4. Preparation of YP-COS Stabilized W_1_/O/W_2_ Emulsions

**Figure 1:**
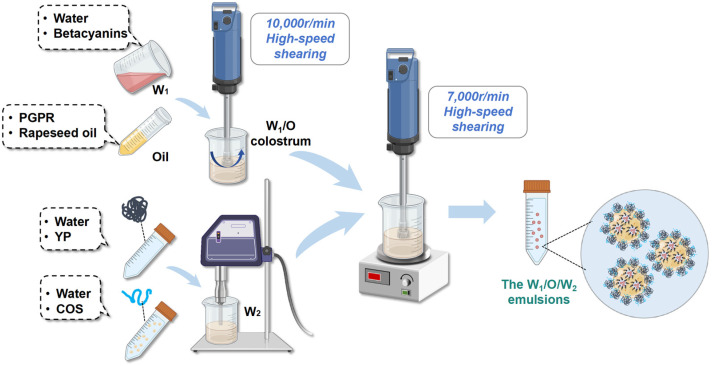
The schematic diagram of the preparation process of the W_1_/O/W_2_ emulsions.

The YP-COS stabilized W_1_/O/W_2_ emulsions containing betacyanins were synthesized through a sequential emulsification process involving two distinct phases [19]. The fabrication process initiated with constructing a primary water-in-oil (W_1_/O) emulsion, subsequently dispersed within the optimized YP-COS aqueous phase (W_2_) to form the final triple-phase architecture, as schematized in Figure 1. The process of preparing W_1_/O colostrum was systematically incorporated in Section 2.3. A precisely measured 1.2 mL W_1_ phase was combined with 10.8 mL oil phase (1:9, *v*/*v*) in sterile centrifuge tubes, through high-energy homogenization, resulting in a stable W_1_/O colostrum. The colostrum was systematically incorporated into three W_2_ phase formulations (YP (2%)-COS (6%), YP (1.25%)-COS (3.75%), and YP (0.5%)-COS (1.5%), *w*/*v*) at volume fractions of 30%, 70%, and 90%. Secondary emulsification through controlled homogenization (7000 rpm, 120 s) generated nine experimental variants, designated as: YP (2%)-COS (6%)-1 to -3, YP (1.25%)-COS (3.75%)-1 to -3 and YP (0.5%)-COS (1.5%)-1 to -3.

### 2.5. Confocal Laser Scanning Microscopy (CLSM)

To observe the distribution of emulsion and protein, the emulsion systems were examined through confocal laser scanning microscopy (CLSM, Leica Stellaris 5, Wetzlar, Hesse, Germany) operating in fluorescence detection mode with a 20× magnification objective. For microscopic analysis, the emulsion specimens were diluted with PBS at a 1:2 (*v*/*v*) ratio before imaging. Subsequently, 1 mL of the diluted sample was combined with 40 µL aliquots of both Nile Blue (0.01%, *w*/*v*) and Nile Red (0.01%, *w*/*v*) fluorescent dyes. This staining protocol enabled simultaneous visualization of different structural elements within the emulsion system [20,21]. After 2 h of dark incubation, the stained samples were mounted onto glass slides and covered with the coverslips to eliminate air gaps. The samples were then allowed to equilibrate for 2 min. Microscopic images were subsequently acquired using specific excitation wavelengths, with fluorescence detection performed at 488 nm and 633 nm.

### 2.6. Microstructural Observation

The emulsion specimens from nine experimental groups underwent a 10-fold dilution with deionized water prior to microscopic examination. For sample preparation, 20 μL aliquots of the diluted emulsion systems were transferred onto microscopy-grade glass slides (25 × 75 mm) and covered with coverslips (18 × 18 mm) with uniform pressure to achieve monolayer dispersion while minimizing structural deformation and bubble entrapment. Microstructural characterization was performed using an inverted optical microscope (Leica DM1000 LED, Wetzlar, Hesse, Germany) equipped with a 40× magnification objective and digital imaging capabilities. Bright-field illumination was employed to visualize and document the morphological features of the W_1_/O/W_2_ emulsion systems, with particular attention given to maintaining optimal sample thickness and minimizing optical artifacts during image acquisition.

### 2.7. ζ-Potential and Particle Size Measurement

The colloidal properties of emulsion formulations were systematically characterized following standardized preparation protocols. Prior to analysis, all samples were diluted 100-fold with deionized water (pH 7.0) using vortex mixing to ensure uniform dispersion while minimizing multiple scattering effects from lipid components. Hydrodynamic diameter and surface charge characteristics were quantified using a commercial ζ-potential analyzer (Zetasizer Nano ZS, Malvern Panalytical, UK) under a controlled temperature (25 °C). To ensure measurement accuracy, all samples were subjected to a 60-s thermal equilibration period before analysis, followed by zeta potential determination via phase analysis light scattering, and particle size distribution analysis through dynamic light scattering. Triplicate measurements were performed for each sample, with results expressed as mean ± standard deviation (*n* = 3). This rigorous approach ensured *p* < 0.05 coefficient of variation between replicates, confirming measurement reproducibility.

### 2.8. Determination of the Creaming Index

The creaming stability of emulsion systems was quantitatively evaluated through phase separation monitoring using an established methodology [22]. To assess the creaming index (CI) values, equal volumes of all freshly prepared emulsions were transferred into 5 mL glass tubes (inner diameter = 2.2 cm, length = 5.2 cm) and subjected to refrigerated storage at 4 ± 0.5 °C for 3 days to evaluate their stability under chilled conditions. The emulsion samples were periodically photographed under natural light using a digital camera. Immediately after photography, the height of each sample was measured with a ruler. At predetermined time intervals, the heights of the upper emulsifying layer (H_1_, H_2_, H_3_) and the initial height were measured and substituted into the following formula for calculation:

CI (%) = H_storage_/H_0_ × 100 (1)

H_storage_ was the height of the upper emulsifying layer of the emulsions after storing, and H_0_ was the initial height.

### 2.9. Stability Analysis

#### 2.9.1. Thermal Stability Analysis

The thermal degradation kinetics of betacyanins were evaluated across four defined temperature regimes (25, 40, 60, and 80 °C) using a comparative experimental design. The samples included two comparative groups: (1) betacyanins aqueous solution control and (2) optimized W_1_/O/W_2_ emulsion from prior formulation screening. Both groups maintained identical betacyanin concentrations to ensure a valid comparative analysis of thermal stability characteristics. These emulsions were heated in a water bath for 30 min, protected from light. The optical properties of the samples under various thermal conditions were quantitatively analyzed using a UV-Vis spectrophotometric system (Agilent 8453, Santa Clara, CA, USA) at a characteristic wavelength of 535 nm, and the absorbance values were systematically recorded to assess the thermal stability profiles of the samples [23].

#### 2.9.2. Photostability Analysis

The light stability profiles of betacyanin formulations were comparatively assessed using two distinct irradiation regimes. Building upon the optimized W_1_/O/W_2_ emulsion system identified through prior characterization, experimental groups were designed with matched betacyanin concentrations: (1) betacyanin aqueous solution control and (2) an emulsion-encapsulated system. The samples were irradiated under both natural light and UV light (254 nm). Photodegradation kinetics were monitored at 2-h intervals (0–8 h) with triplicate samples per condition. Betacyanin integrity was tracked via a UV-Vis spectrophotometer (Agilent 8453, Santa Clara, CA, USA), and the absorption of the samples was recorded at the peak of 535 nm.

#### 2.9.3. The pH Stability Analysis

The pH-dependent stability of betacyanin formulations was systematically investigated across pH gradients of 3.0, 5.0, 7.0, 9.0, and 11.0 with 0.1 M HCl/NaOH, maintaining ±0.05 pH unit accuracy via pH meter calibration. Aliquots of (a) free betacyanin solution and (b) optimized W_1_/O/W_2_ emulsion were dispersed in pre-equilibrated buffers (25 °C). The samples were placed under different pH conditions while avoiding light exposure. The samples were thoroughly homogenized using a vortex mixer (SCILOGEX, MX-S, Nanjing, China) for 20 min. Post-homogenization samples underwent immediate spectral acquisition, and the absorbance of each sample was measured at 535 nm.

#### 2.9.4. Storage Stability Analysis

The W_1_/O/W_2_ emulsions and betacyanin aqueous solution were transferred to serum bottles and stored in an incubator at 25 °C for 1 to 7 days. Following the storage period, an equal volume of each sample was mixed with hexane to extract the betacyanin-containing aqueous phase. The absorbance of the extracted solutions after various treatment durations was measured using a UV-Vis spectrophotometer (Agilent 8453, Santa Clara, CA, USA), at 535 nm. The retention rates of betacyanins were all calculated using the following formula:

Retention rates (%) = A_e_/A_0_ × 100 (2)

A_e_ was the absorbance value of betacyanins after the above treatment of the stability, and A_0_ was the initial absorbance value of betacyanins.

### 2.10. In Vitro Gastrointestinal Digestion

#### 2.10.1. Simulated Gastric Digestion

The simulated gastric fluid (SGF) was prepared by dissolving 2.0 g NaCl, adding 7.0 mL of 6 mol/L concentrated hydrochloric acid (37%, *w*/*w*) and 3.2 g pepsin, and adjusting the pH to 1.2 using 1.0 M HCl [24]. Freshly prepared emulsions were mixed with an equal volume of pre-warmed SGF (37 ± 0.5 °C). Separately, an equal volume of aqueous betacyanins was combined with the SGF, and the mixture was adjusted to a pH of 2.0 at 37 °C using 1.0 M NaOH. Both sets of samples were incubated in a constant-temperature shaker (Zhi in, ZWY-2102C, Shanghai, China) at 37 °C and 100 rpm. The experiment required five sampling sessions at 0, 30, 60, 90, and 120 min, respectively. After digestion, the pH was raised to 7.0 with 0.25 M NaOH to inactivate pepsin. Betacyanins were extracted using an ethanol/n-hexane (1:2, *v*/*v*) mixture. The solution was shaken and allowed to settle for approximately 3 min; betacyanins accumulated in the bottom layer. 600 μL of the bottom layer was measured at 535 nm using a multimode microplate reader (BioTek Epoch2, Winooski, VT, USA) to evaluate gastric digestion efficiency.

#### 2.10.2. Simulated Intestinal Digestion

After simulated gastric digestion, the remaining samples were adjusted to pH 7.0 with 0.25 M NaOH to inactivate pepsin, terminating the gastric phase and facilitating the subsequent transition to the intestinal digestion stage. In vitro intestinal digestion simulations were conducted using a modified standardized protocol [24]. The preparation of simulated intestinal fluid (SIF) involved two sequential steps: initially, 1.7 g potassium dihydrogen phosphate and 0.97 g bile salt extract were dissolved in 125 mL deionized water and pH-adjusted to 7.0 with 0.25 M NaOH. Separately, 2.5 g of pancreatin was dissolved in distilled water. The two solutions were then quantitatively combined and diluted with deionized water to achieve a final volume of 250 mL and maintained pH at 7.0, ensuring proper enzymatic activity and ionic strength for the digestion simulation. After two hours of gastric digestion and pepsin inactivation, samples were subjected to intestinal phase analysis. Each test solution was combined with an equivalent volume of SIF and incubated in a thermostatic orbital shaker (Zhi in, ZWY-2102C, Shanghai, China) at 37 ± 0.5 °C and 100 rpm. Aliquots were collected at 0, 30, 60, 90, and 120 min, extracted with ethanol/hexane (1:2, *v*/*v*), and analyzed spectrophotometrically at 535 nm using a multi-mode microplate reader (BioTek Epoch2, Winooski, VT, USA) to quantify bioactive component concentrations and calculate retention rates throughout the digestion process. The retention rates of betacyanins were calculated using the following formula:

Retention rates (%) = A_d/_A_0_ × 100 (3)

A_d_ represented the absorbance after a fixed time of digestion and A_0_ represented the absorbance of the initial digestion.

### 2.11. Experimental Data Analysis and Processing

Unless otherwise stated, all experiments were repeated three times. The data obtained were analysed using SPSS software (25.0), and Origin (9.0) was used for graphing. Statistical differences between samples were analyzed using Duncan’s multiple comparison test, with statistical significance defined at *p* < 0.05.

## 3. Results and Discussions

### 3.1. Confocal Laser Scanning Microscopy (CLSM)

**Figure 2:**
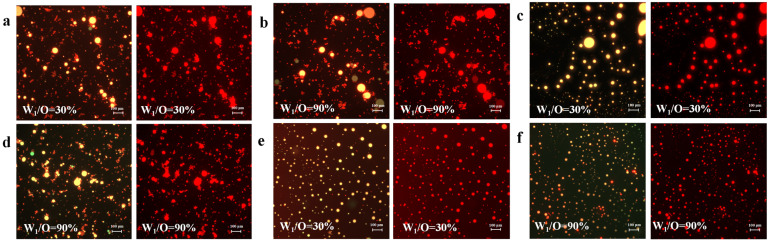
CLSM micrographs of the W_1_/O/W_2_ emulsions. (**a**,**b**) YP (0.5%)-COS (1.5%); (**c**,**d**) YP (1.25%)-COS (3.75%); (**e**,**f**) YP (2%)-COS (6%). Scale bar: 100 μm.

The phase distribution analysis of the oil phase constituents and YP-COS complexes was quantitatively mapped using confocal laser scanning microscopy (CLSM). Results in Figure 2 present double and single-channel images of protein and polysaccharide complexes. The presence of a small number of droplets showing a green color in Figure 2d is lipids stained green by Nile Red. YP constituents were selectively stained with Nile Blue, producing red fluorescence, as demonstrated in Figure 2. The distribution pattern and stain aligned with the processing results of Zhang et al. [25]. Monochromatic analysis of the red channel provided interfacial topology, unequivocally demonstrating the concentric arrangement of YP-COS complexes at droplet interfaces. This distinctive annular configuration not only validates successful emulsion fabrication but also demonstrates interfacial YP-COS complexation through interactions, as corroborated by the previous study [9]. The surface of the colostrum was tightly encapsulated by the YP-COS film, while excess complexes maintained colloidal dispersion in the continuous aqueous phase [26]. The YP-COS conjugates formed a cohesive interfacial film on emulsion droplets, thereby reducing surface tension, inhibiting droplet coalescence, and improving the stability of the droplet. The oil droplet (green) and YP-COS (red) were overlapped to show the formation of the W_1_/O/W_2_ emulsions (yellow). The aqueous phase (black) inside the oil droplet was not stained due to the blocking of the oil shell, rendering it unobservable.

From Figure 2, it is evident that the diameter of the droplet gradually decreased when the concentration of YP-COS kept increasing. Figure 2e,f show that the droplet size of the emulsions remained relatively small, regardless of whether the colostrum percentage of 30% or 90% when the W_2_ concentration was YP (2%)-COS (6%). This suggests the structural property of proteins and polysaccharides that played a role in enhancing the stability of the system. At the high concentration of YP (2%)-COS (6%), YP and COS interactions were increased. This was in agreement with the previous results [25]. Therefore, the emulsions with YP (2%)-COS (6%) demonstrate superior properties.

### 3.2. Microstructure and Appearance

**Figure 3:**
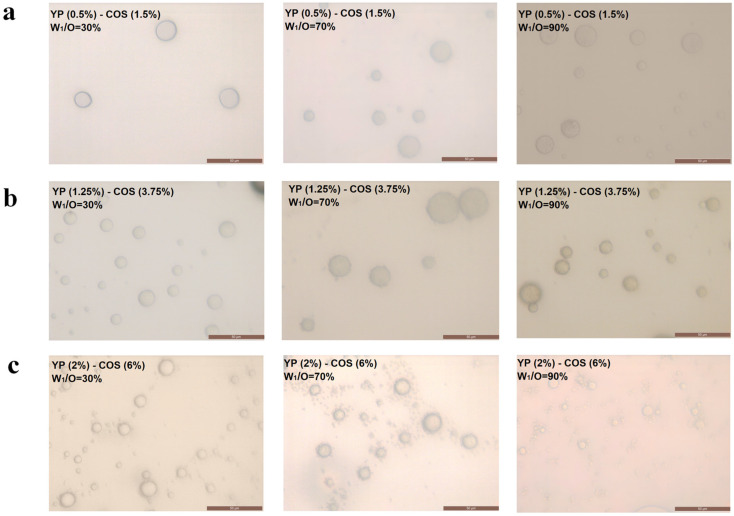
The microscope images of the W_1_/O/W_2_ emulsions. (**a**) YP (0.5%)-COS (1.5%); (**b**) YP (1.25%)-COS (3.75%); (**c**) YP (2%)-COS (6%). From left to right, W_1_/O colostrum percentages are 30%, 70%, and 90% in order. Scale bar: 50 μm.

To enhance structural characterization, all emulsion specimens were appropriately diluted prior to microscopic analysis. Figure 3 demonstrates homogenized emulsions containing uniformly dispersed aqueous microdomains. Interfacial regions exhibited distinctive fibrillar protrusions from spherical globules, corresponding to the YP-COS complexes at the oil-water interface. The central region of the oil phase contained discrete microdomains containing betacyanin compounds, whose morphology aligned with theoretical predictions. The formation of small and compact clusters among droplets can be attributed to the accumulation of proteins at the interface due to the electrostatic binding of COS to multiple YPs simultaneously, which acted as a bridging link between the emulsion droplets to form a three-dimensional network structure. Conversely, systems lacking robust YP-COS interactions exhibited phase-segregated configurations: amphiphilic YPs localized at interfaces while hydrophilic COS remained dispersed in aqueous phases, eliminating polysaccharide-mediated bridging. This led to the dispersion of the emulsion droplets [27]. These microscopic observations confirmed that the double emulsion system maintained the characteristic structural organization of W/O/W emulsions, with the internal aqueous phase (W_1_) forming densely packed domains within the oil globules, uniformly themselves dispersing throughout the external aqueous continuum (W_2_) [28]. The emulsion droplets were approximately spherical and surrounded by a layer of the outer YP-COS complex solution, the middle oil phase, and the inner betacyanin aqueous solution, indicating the successful distribution of the W_1_/O/W_2_ emulsions. Figure 3c exhibits the optimal W_1_/O/W_2_ emulsion formulation with YP (2%)-COS (6%) and a 90% W_1_/O colostrum achieved minimal particle size (3.0 μm), showing superior structural integrity.

### 3.3. Average Particle Size

**Figure 4:**
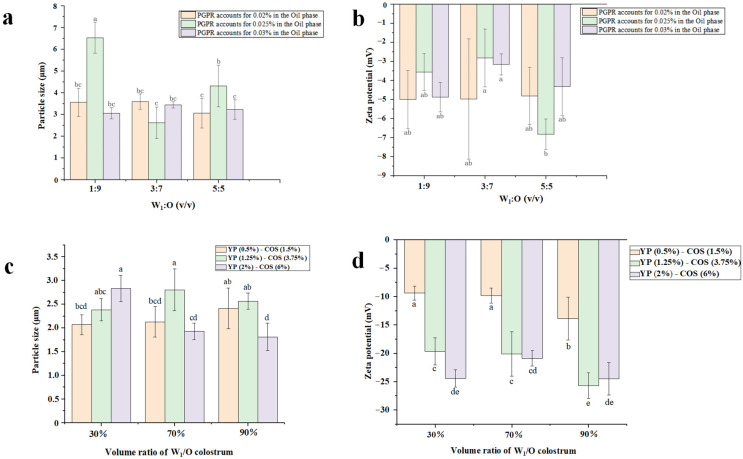
Particle size and potential of different ratios of the W_1_/O colostrum and the W_1_/O/W_2_ emulsions. (**a**,**b**) Particle size and zeta potential of the W_1_/O colostrum at different ratios and (**c**,**d**) Particle size and zeta potential of the W_1_/O/W_2_ emulsions at different ratios. The data are expressed as mean ± standard deviation (*n* = 3). The presence of a letter (a–e) situated above or below the error bars signifies a significant discrepancy between the means (*p* < 0.05).

In emulsion systems, dispersed phase dimensions critically determine physicochemical and functional attributes, with mean droplet diameter and polydispersity index governing optical characteristics, rheological profiles, and colloidal stability. Within the context of food applications, these dimensional parameters played a pivotal role in determining product stability during storage and sensory characteristics, particularly influencing textural perception and flavor release profiles [26]. The inverse correlation between emulsion stability and droplet diameter arises from gravitational separation dynamics, where reduced droplet dimensions significantly suppress creaming/sedimentation through enhanced Brownian motion dominance over gravitational forces [29]. However, due to the structural complexity of W_1_/O/W_2_ emulsions, this metric reflected stability only to a certain extent. Figure 4a reveals the W_1_/O colostrum containing 0.03% (*v*/*v*) PGPR exhibited relatively smaller particle sizes and little data volatility regardless of the water-oil ratio, validating a prior optimization study [30]. When the water-oil ratio was 3:7 (*v*/*v*), the colostrum with 0.025% (*v*/*v*) PGPR had the smallest particle size (2.62 ± 0.73 μm), showing no significant difference (*p* > 0.05). The colostrum with 0.03% (*v*/*v*) PGPR and water-oil ratio of 1:9 (*v*/*v*) exhibited the second minimal particle size (3.05 ± 0.27 μm)**,** presenting a significant difference (*p* < 0.05). The data suggest that a suitable system for further experiments could be colostrum with a water-oil ratio of 1:9 and a PGPR of 0.03% (*v*/*v*).

Results in Figure 4c present the emulsions with YP (2%)-COS (6%) as W_2_ and a 90% volume ratio of the W_1_/O colostrum achieved unprecedented size reduction (1.81 ± 0.29 μm), showing a significant difference compared to the other groups (*p* < 0.05). The particle size result was similar to that of the optical microscope result described above, indicating the W_1_/O/W_2_ emulsion formulation with YP (2%)-COS (6%) and a 90% W_1_/O colostrum achieved minimal particle size, and the emulsion particle size was in the micro range. Moreover, the particle dimensions of the dual-phase emulsion were observed to be on the same order of magnitude as those reported for betacyanins-encapsulated W/O/W systems in the previous study [31]. The particle size of the W_1_/O/W_2_ emulsions had been significantly decreased compared to the W_1_/O colostrum, confirming enhanced kinetic stability mechanistically attributed to YP-COS interfacial engineering. Summarily, the smallest particle size is concluded to be the emulsion with YP (2%)-COS (6%) and 90% W_1_/O colostrum.

### 3.4. Zeta-Potential

In addition to particle size distribution, zeta-potential is also a crucial parameter for characterizing the stability of emulsions. As a significant metric for surface characterization, zeta-potential could be used to compare differently charged surfaces, regardless of the pressure, flow conditions, or electrolyte used for measurement [32]. The charge intensity of emulsion droplets is vital for determining emulsion stability and functionality, with higher absolute zeta-potential values indicating greater system stability [33]. As shown in Figure 4b, the highest absolute zeta-potential value of 6.83 ± 0.80 mV was observed in emulsions with 0.025% (*v*/*v*) PGPR and a water-oil ratio of 5:5 (*v*/*v*), while this was not significantly different from other groups (*p* > 0.05). When the water-oil ratio was 1:9 (*v*/*v*), the potential values were overall high. With a water-oil ratio of 1:9 (*v*/*v*), the zeta potentials were 5.00 ± 1.51 mV (second highest values) and 4.88 ± 0.83 mV (fourth highest value), corresponding to emulsions with 0.02% (*v*/*v*) PGPR and 0.03% (*v*/*v*) PGPR, respectively. Impurities in the same oil phase, such as phospholipids, free fatty acids, or mineral ions, could affect zeta-potential magnitude due to volume differences [34]. Moreover, when the water-oil ratio was maintained at 1:9 (*v*/*v*), the measured potentials across all tested PGPR ratios exhibited no significant difference (*p* > 0.05). This suggests that adjusting PGPR concentrations has a limited impact on the stability of the W_1_/O-type colostrum emulsion under the water-oil ratio of 1:9 (*v*/*v*). Combined with the aforementioned particle size data, these findings suggest that maintaining a water-oil ratio of 1:9 (*v*/*v*) is a robust parameter for optimizing the preparation of colostrum oil phase formulations.

The zeta potential values for various W_1_/O/W_2_ emulsion ratios are presented in Figure 4d, all of which were negative, indicating negatively charged emulsion droplets. From Figure 4d, with a 90% colostrum volume fraction in the W_1_/O colostrum, the emulsions containing YP (1.25%)-COS (3.75%) in the external aqueous phase had the highest absolute potential value of 25.72 ± 2.26 mV, followed by those with YP (2%)-COS (6%) at 24.51 ± 2.84 mV. These values were comparable to those reported by Harimurti et al. for water-in-oil nanoemulsions containing curcumin and betacyanins [35]. The zeta potential analysis revealed a significant difference between the two systems, with the W_1_/O/W_2_ emulsions exhibiting higher absolute zeta potential values than the W_1_/O colostrum system. For W_1_/O/W_2_ emulsions with 90% W_1_/O colostrum, smaller particle sizes and absolute potential values indicated superior stability. Although the YP (1.25%)-COS (3.75%) emulsions had higher absolute potential values than those with YP (2%)-COS (6%), considering both particle size and zeta-potential, the W_1_/O/W_2_ emulsions with YP (2%)-COS (6%) and 90% colostrum demonstrate the highest stability.

### 3.5. Creaming Index

**Figure 5:**
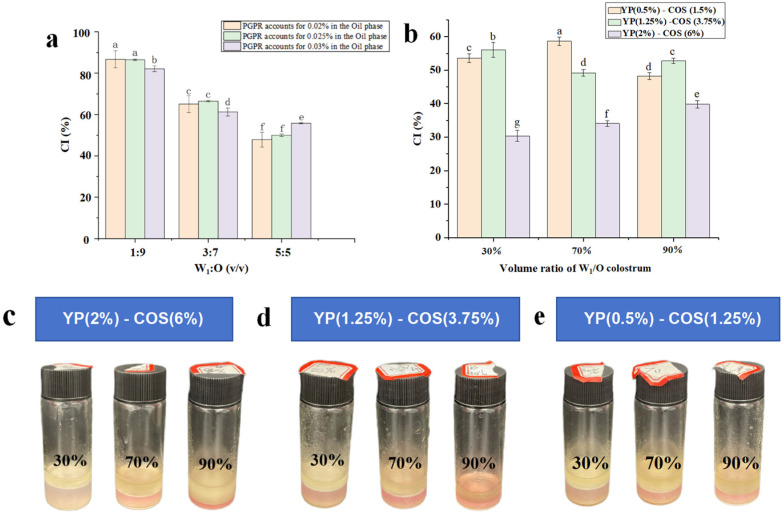
(**a**) the CI of the W_1_/O colostrum with different ratios and (**b**) the CI of the W_1_/O/W_2_ emulsions (30%, 70%, 90% as volume ratio of the W_1_/O colostrum). The data are expressed as mean ± standard deviation (*n* = 3). (**c**) YP (2%)-COS (6%) post-storage appearance patterns; (**d**) YP (1.25%)-COS (3.75%) post-storage appearance patterns; (**e**) YP (0.5%)-COS (1.25%) post-storage appearance patterns. The presence of a letter (a–g) situated above the error bars signifies a significant discrepancy between the means (*p* < 0.05).

The creaming index (CI), a quantitative measure of emulsion phase separation, exhibited an inverse relationship with stability. The elevated CI values indicate compromised structural integrity [31]. From the above results of particle size and zeta-potential, the ratios of PGPR were screened from the water-oil ratio of 1:9 (*v*/*v*). Emulsions exhibited the largest CI when the water-oil ratio was 1:9 (*v*/*v*) and PGPR was 0.02% (*v*/*v*), resulting in relative instability. The CI values exhibited minimal variation across different aqueous-oil phase ratios, suggesting limited formulation-dependent interfacial activity. Notably, the W_1_/O colostrum containing 0.03% (*v*/*v*) PGPR demonstrated significantly reduced CI values (*p* < 0.05) in Figure 5a. When the water-oil ratio was 1:9 (*v*/*v*), it was normal that the CI may be larger than the other groups due to the higher ratio of the oil phase [36]. Combining the experimental results of particle size and potential, critical formulation optimization identified 0.03% (*v*/*v*) PGPR with a 1:9 (*v*/*v*) water-oil ratio as the stability-optimal configuration. Therefore, this formulation was selected for the W_1_/O/W_2_ emulsions for the experiments.

The W_1_/O/W_2_ emulsion samples maintained at 4 °C demonstrated storage time-dependent destabilization (Figure 5c–e). After the 3-day storage period, the emulsions typically exhibited increasing instability with prolonged storage time, ultimately leading to phase separation. As evidenced in Figure 5d,e, the phase separation behavior of W_1_/O/W_2_ emulsions prepared with different W_1_/O colostrum volume ratios exhibited no significant variations when the concentration of complexes was YP (0.5%)-COS (1.5%) or YP (1.25%)–COS (3.75%). The YP-COS complex demonstrated concentration-dependent stabilization efficacy, with superior emulsion stability achieved at high concentrations of YP (2%)-COS (6%). As illustrated in Figure 5b, the emulsions with YP (2%)-COS (6%) and a 30% volume share W_1_/O colostrum exhibited the minimum CI value of 30.35% ± 0.02, but the 30% share was less effective in color protection. Comparative analysis confirmed that the W_1_/O/W_2_ emulsions utilizing the YP (2%)-COS (6%) complex as the external aqueous phase displayed significantly lower creaming indices (*p* < 0.05) and enhanced macroscopic stability relative to other groups. Therefore, this formulation is established as the optimal balance between physical stability and functional performance. Combining the above experimental results, the W_1_/O/W_2_ emulsions containing YP (2%)-COS (6%) and 90% W_1_/O colostrum are the most stable emulsion systems suited for the subsequent experiments.

### 3.6. Stability Analysis

#### 3.6.1. Thermal Stability

**Figure 6:**
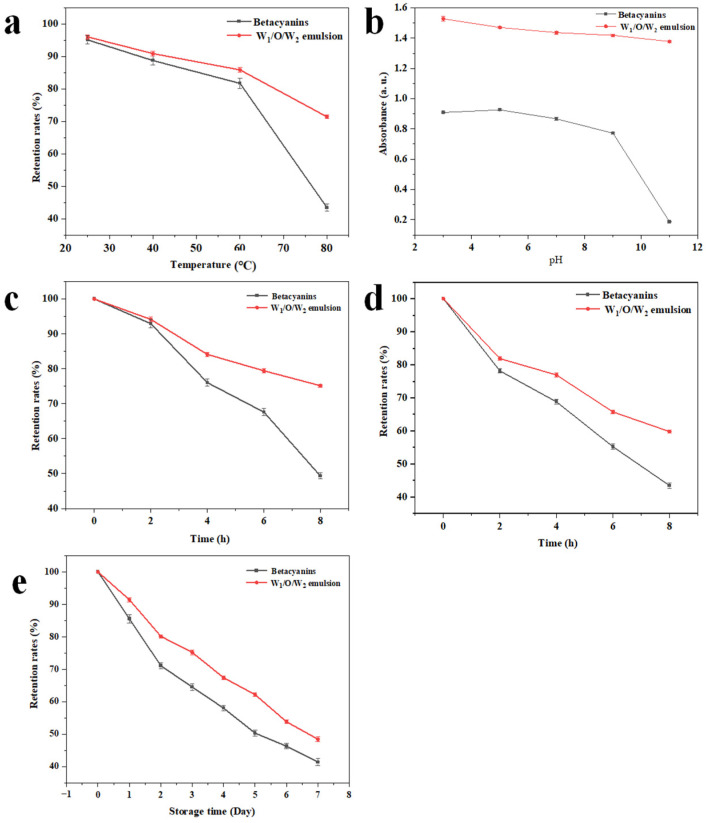
(**a**) comparison of retention rates change between the W_1_/O/W_2_ emulsions and betacyanins aqueous solutions under different temperature treatments; (**b**) absorbance of the W_1_/O/W_2_ emulsions and betacyanins aqueous solutions under different pH treatments; (**c**) comparison of retention rates change between the W_1_/O/W_2_ emulsions and betacyanins aqueous solutions under natural light treatment; (**d**) comparison of retention rates change between the W_1_/O/W_2_ emulsions and betacyanins aqueous solutions under UV treatment (254 nm); (**e**) comparison of retention rates change between the W_1_/O/W_2_ emulsions and betacyanins aqueous solutions under storage for seven days.

Most food processing methods involve heat treatment at various stages to inactivate or reduce microbial content, remove moisture, inactivate enzymes, and induce physical or chemical changes, ensuring that consumers receive safe and palatable foods. Thus, the thermal stability of betacyanins, commonly used as food coloring agents, is crucial during heat treatments that ensure food safety and palatability [37]. To investigate the emulsion stability over a range of temperatures, the absorbances of the emulsions were measured against the betacyanins solution after subjecting them to various temperatures of heat treatment, and the retention rates were calculated and compared (Figure 6a). The retention rates specifically reflected the residual betacyanin remaining within the emulsion, excluding any released into the surrounding medium. At 25 °C, the retention rates of the two samples were nearly equal and close to 95%, which declined with increasing temperature. Notably, emulsion-encapsulated betacyanins consistently exhibited higher retention rates than aqueous solutions. Furthermore, the retention rate of betacyanins in the W_1_/O/W_2_ emulsions treated at 80 °C for 30 min was significantly higher than that of betacyanins in the dissolved state (*p* < 0.05). This finding suggests that the thermal stability of betacyanins after emulsion encapsulation has been improved. This enhanced thermal stability was attributed to the protective W_1_/O/W_2_ emulsion structure and the YP-COS network. Supporting this, it has been found that polysaccharide-based multilayer emulsions can maintain the physical stability of β-carotene for close to 60 min in heat treatments up to 121 °C [38]. This suggests that the thermal stability of emulsion-embedded bioactives can be significantly improved. Thus, YP-COS-stabilized W_1_/O/W_2_ emulsions effectively improve betacyanin thermal stability during food processing.

#### 3.6.2. The pH Stability

Many natural colorants exhibit a wide range of variability in response to pH changes, limiting their applications in multiphase food coloring systems. The stability of betacyanins is highly pH-dependent, causing their reddish-purple color to shift toward violet at lower pH or yellow at higher pH [39]. Under aerobic conditions, betacyanins are the most stable at a pH of about 5.5 [40]. As illustrated in Figure 6b, the betacyanin aqueous solution had maximum absorbance at a pH of around 5.0. The absorbance of the pigment demonstrated relative constancy between pH 3 and 6, implying that betacyanins maintained stability within this pH range. This finding was supported by previous research on betacyanin pH stability [41]. However, the absorbance of the betacyanin aqueous solution dropped sharply after the pH reached 9.0. In contrast, within the W_1_/O/W_2_ emulsions, the absorbance remained largely unchanged across a broader pH spectrum, continuing up to pH 11.0. The underlying mechanism for this difference can be attributed to the changes in electrostatic interactions as the pH increased. Initially, there was electrostatic repulsion between YP and COS. As pH increased, YP and COS electrostatic repulsion turned to attraction. This shift facilitated the formation of YP-COS non-covalent complexes. These complexes played a crucial role in stabilizing emulsion droplets by enhancing interfacial interactions. Concurrently, the intensified electrostatic forces promoted structural compaction of both the complexes and the emulsion system [42]. Consequently, the absorbance of the emulsion remained relatively stable across varying pH conditions. This stability suggests that the emulsion structure effectively protected the retention of betacyanins.

#### 3.6.3. Photostability

Pigments are typically unstable under light conditions. When betacyanins are exposed to UV or visible light irradiation, their chromophore group’s electrons become excited to a higher energy state. This increases the betacyanin molecule’s reactivity and reduces its activation energy. To prevent interference from other emulsion media on betacyanin absorbance, the experimental extraction volume was set at 6 mL. Both dissolved betacyanins and those within the emulsions exhibited varying degrees of degradation following exposure to natural light and UV irradiation, as illustrated in Figure 6c,d. The 100% value represented the initial betacyanin amount in the emulsion at t = 0, serving as the reference for calculating retention rates. The initial absorbance was measured immediately after sample preparation so that the retention rates of both samples were approximately 100% at 0 h. The retention rates of emulsion-contained betacyanins were significantly higher than those in the dissolved state after natural light treatment (*p* < 0.05). Similarly, under UV irradiation, the retention rates of emulsion-contained betacyanins were reduced and were significantly higher (*p* < 0.05) than those in the dissolved state. The comparison of retention rates indicates that natural pigments are prone to light-induced degradation, causing color loss. In contrast, emulsion-embedded betacyanins exhibit enhanced stability, possibly attributable to macroscopic mediators that modify the pigment molecules and improve stability [43]. Summarily, based on the mechanical strength of the interfacial YP-COS complex shell and oil phases, the emulsions have superior stability against treatments like heating, pH shift, and light.

#### 3.6.4. Storage Stability

Storage stability is crucial for pigments used primarily as colorants. After 7 days of storage, the W_1_/O/W_2_ emulsions show superior retention rates in Figure 6e compared with the betacyanin solution. This observation indicated that the absorbance of betacyanins encapsulated in the YP-COS-based W_1_/O/W_2_ emulsions experienced a smaller absorbance reduction than dissolved betacyanins. COS adsorption increased steric hindrance at the YP-stabilized oil-water, so the emulsion structure stabilization was effectively improved. The enhanced spatial resistance likely restricted molecular mobility, improving interfacial stability, and thereby offering a protective effect on betacyanin integrity under varying environmental conditions [44]. The enhanced storability may stem not solely from the emulsion’s protection but also from the combined stability of betacyanins and the W_1_/O/W_2_ emulsions. Betacyanins tend to form monodisperse droplets with a homogeneous size distribution, potentially due to increased electrostatic repulsion between droplets and higher potential barriers against agglomeration and flocculation [45]. In line with these findings, prior studies have shown that gelatin-chitosan-betaine complexes exhibit considerable potential as stabilizers for high internal phase emulsions (HIPEs) in the fabrication of 3D-printed functional foods. The finding indicates that chitosan-based composite matrices could improve betacyanins’ stability, potentially through optimized interfacial interactions within HIPE architectures [46]. Furthermore, Liu et al. have demonstrated that natural pigments encapsulated in chitosan (CS)-NaOH-modified casein nanoparticles (CA NPs) exhibited enhanced long-term stability, which was attributed to the robust emulsifying properties of the composite carrier system [47]. The emulsion, affected by various external factors, still retard the release of betacyanins. This contributes to prolonging color development, product shelf-life, and maintaining antioxidant properties.

### 3.7. In Vitro Digestibility of the W_1_/O/W_2_ Emulsions

**Figure 7:**
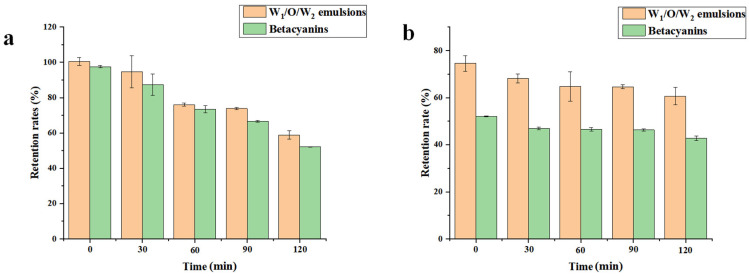
(**a**) Comparison of retention rates of emulsions and single betacyanins during simulated gastric digestion and (**b**) comparison of retention rates of emulsions and single betacyanins in simulated intestinal digestion.

Figure 7a illustrates the retention rates of betacyanins in the W_1_/O/W_2_ emulsions during in vitro gastrointestinal digestion. During the initial gastric phase, betacyanin concentration in the digestive fluid peaked irrespective of encapsulation status. This observation underscores the transient bioavailability window of phytochemicals during early digestion. The retention rate of unencapsulated betacyanins was 97.49%, while the retention rate of betacyanins encapsulated within the emulsions was 99.91%. Pronounced betacyanin degradation was observed during the gastric phase digestion, mechanistically linked to their structural stability optimum (pH 5.5). This phenomenon was exacerbated under simulated gastric conditions (pH 2.0), where progressive molecular destabilization occurred, ultimately compromising structural integrity. The rapid decline in detectable betacyanins upon entering the intestinal digestion stage was primarily due to the neutral pH environment of the intestine, which facilitated further degradation of betacyanins [48]. The emulsion-encapsulated betacyanins demonstrate enhanced retention and stability throughout the gastric phase, highlighting the encapsulation system’s capacity to preserve bioactive compound integrity during gastrointestinal transit. This protective mechanism effectively delays the bioactive release until intestinal conditions.

In Figure 7b, a decrease in the retention rates of betacyanins within the W_1_/O/W_2_ emulsions was observed as the digestion process transitioned to the enteric phase. In the final stage of intestinal digestion, non-encapsulated betacyanins exhibited markedly lower retention (41.42%) compared to emulsion-encapsulated counterparts (58.39%), demonstrating the protective efficacy of encapsulation in maintaining bioactive integrity throughout gastrointestinal transit. The main reason may be that betacyanins were isolated from the external aqueous phase in the W_1_/O/W_2_ emulsions, thereby minimizing contact with the digestion solution [24]. The study has shown that COS significantly reduced the digestibility of proteins, which allowed YP-COS complexes to establish hierarchical protection for the encapsulated betacyanin payload through dual-phase stabilization mechanisms [49]. The interfacial dynamics of multiphase systems created steric hindrance mechanisms that impeded critical digestive processes, thereby prolonging the retention period of entrapped bioactive agents within colloidal carriers. Additionally, the study has reported that the rational design of the hydrophilic domains using renewable biopolymeric materials demonstrated the feasibility of modulating the release kinetics of bioactive payloads. Controlled or sustained release of emulsion-encapsulated functional ingredients can be achieved by incorporating natural biopolymers into the aqueous phase [50]. The emulsions modulated betacyanin release kinetics and extended their biological residence, thereby enhancing the compounds’ capacity to holistically exert antioxidant, anticancer, and anti-inflammatory therapeutic efficacy through sustained bioactivity maintenance. Collectively, these experimental findings demonstrate that YP-COS-stabilized W_1_/O/W_2_ emulsions serve as functional delivery vehicles, enhancing betacyanin bioaccessibility through targeted gastrointestinal protection and controlled release mechanisms.

## 4. Conclusions

This study demonstrates that the W_1_/O/W_2_ emulsions constructed with YP-COS effectively encapsulated betacyanins and enhanced emulsion stability. The mechanical strength of the interfacial YP-COS complex shell and oil phases contributes to the emulsion’s superior stability against treatments such as thermal treatment, photolytic exposure, pH gradients, and extended storage compared to betacyanin aqueous solution. However, the long-term stability of these emulsions for specific applications in real products requires further investigation. The in vitro intestinal lipid digestion results indicate that pigment retention in the emulsions is significantly improved and that the emulsions delay the release of betacyanins, thereby facilitating their role in the human body. The findings of this experiment support the broader application of betacyanins in the fields of food, cosmetics, and dyes while providing valuable insights into the interactions between proteins and polysaccharides as emulsion components. This study advances the development of emulsion encapsulation systems for protecting bioactive components and demonstrates application prospects for YP-COS emulsions in food formulations such as ice cream, beverages, yogurt, and fat replacers. Nonetheless, the long-term stability and action mechanisms of YP-COS-stabilized emulsions warrant further exploration to fully realize their potential in commercial applications.

## Data Availability

The original contributions presented in this study are included in the article. Further inquiries can be directed to the corresponding authors.
